# Current limits for flowmeter resistance in metabolic carts can negatively affect exercise performance

**DOI:** 10.14814/phy2.14814

**Published:** 2021-04-27

**Authors:** Fernando G. Beltrami, Jérôme Kurz, Elena Roos, Christina M. Spengler

**Affiliations:** ^1^ Exercise Physiology Lab Institute of Human Movement Sciences and Sport ETH Zurich Zurich Switzerland; ^2^ Zurich Center for Integrative Human Physiology (ZIHP) University of Zurich Zurich Switzerland

**Keywords:** ergospirometry, exercise testing, flowmeter, resistance, respiratory muscle work, respiratory muscles

## Abstract

**Purpose:**

To investigate whether a metabolic cart using a flowmeter in the upper range of accepted resistance to airflow (<1.5 cmH_2_O∙L^−1^∙s^−1^ for flows up to 14 L∙s^−1^, American Thoracic Society) negatively impacts exercise performance in healthy individuals.

**Methods:**

16 recreationally active males (age 25 ± 1 years, height 180 ± 6 cm, weight 73.5 ± 5.8 kg, all mean ± SD) performed two incremental tests on a bicycle ergometer on each of two visits, using a metabolic cart with a flowmeter of either low (Oxycon Pro) or high (Innocor) airflow resistance. Mouth pressures, gas exchange, blood lactate concentration [La^−^], perception of breathlessness, respiratory, and leg exertion were assessed throughout the tests.

**Results:**

Tests performed with the Innocor were significantly shorter (15.3 ± 3.2 vs. 15.8 ± 3.3 min, *p* < 0.0001) and showed higher maximal flow resistance (1.3 ± 0.2 vs. 0.3 ± 0.0 cmH_2_O∙L^−1^∙s^−1^, *p* < 0.0001). At end‐exercise, peak oxygen consumption (−200 ± 220 ml.min^−1^, *p* < 0.0001), minute ventilation (−19.9 ± 10.5 L.min^−1^, *p* < 0.0001), breathing frequency (−5.4 ± 5.2 breaths.min^−1^, *p* < 0.0001), heart rate (−2.1 ± 3.6 bpm, *p* = 0.002) and [La^−^] (−0.7 ± 1.0 mmol.L^−1^, *p* < 0.0001), but not tidal volume (−0.1 ± 0.2 L, *p* = 0.172) were lower with the Innocor, while the perception of breathlessness was higher (+3.8 ± 5.1 points, *p* < 0.0001).

**Conclusions:**

Airflow resistance in the upper range of current guidelines can significantly affect exercise performance and respiratory pattern in young, healthy males during incremental exercise. The present results indicate the need to revisit guidelines for devices used in ergospirometry.

## Graphical abstract

Current guidelines allow for respiratory resistance up to 1.5 cmH_2_ = L/s in metabolic carts. We demonstrate that equipment operating in the upper limit of this range negatively affect exercise performance. Current guidelines should be re‐considered.

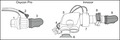

**New findings**

What is the central question of this study?

- Do metabolic carts using technologies to measure airflow during exercise tests that are in the upper limits of current guidelines for airflow resistance negatively affect performance and breathing pattern in young healthy individuals?

What is the main finding and its importance?

- Devices with higher airflow resistance negatively affect performance by increasing the perception of breathlessness and potentially the work of respiratory muscles. The shorter time to exhaustion during an incremental test leads to the underestimation of V̇O_2max_.

## 1 INTRODUCTION

Metabolic carts require a participant to breathe through a mask or mouthpiece which channels the inspired and expired air through different sensors in order to determine respiratory flow, volume, and gas fractions. If commercial equipment meets the requirements for accuracy of measurement and negligible interference, it can be deemed of sufficient quality to be used in research and clinical settings (Macfarlane, 2001). Despite the numerous reports in the literature regarding the validity and reliability of different metabolic carts (Carter & Jeukendrup, 2002; Fontana et al., 2009; Rietjens et al., 2001), substantially less effort has been placed in determining whether they fulfill the requirement of negligible interference. The current guidelines for spirometry recommend that flowmeters produce resistances <1.5 cmH_2_O∙L^−1^∙s^−1^ for flows up to 14 L∙s^−1^ (American Thoracic Society, 1995; Miller, 2005) or <2.5 cmH_2_O∙L^−1^∙s^−1^ for flows up to 14 L∙s^−1^ in “monitoring” devices; the recommendations for cardiopulmonary exercise testing follow the same standard (Weisman, 2003), although it does not specify which of the two resistance limits should be respected. This is a relatively high ceiling, as intentionally loading the respiratory muscles with resistances >3 cmH_2_O∙L^−1^∙s^−1^ during intense cycling is known to affect breathing pattern and exercise performance (Harms et al., 2000).

**FIGURE 1:**
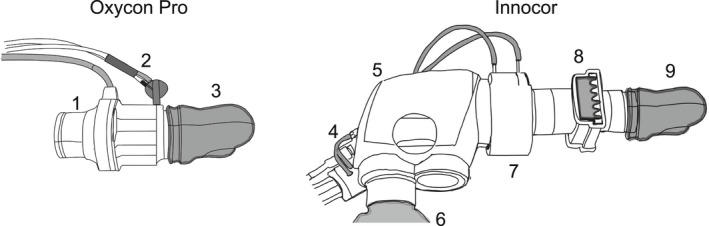
Representation of the set‐up for the Oxycon Pro (left) and Innocor (right). 1: flat fan (called triple V sensor), which is encased by a ring‐shaped sensor and a plastic cover; 2: sample line into the analyzer; 3: mouthpiece (same as used for the Innocor); 4: sample line into the analyzer; 5: rebreathing valve unit with disposable silicon insert inside; 6: rebreathing bag used for cardiac output monitoring; 7: flowmeter; 8: bacterial filter; 9: mouthpiece. Dead‐space is estimated at 35 ml with the Oxycon and 143 ml with the Innocor (including bacterial filter)

**FIGURE 2:**
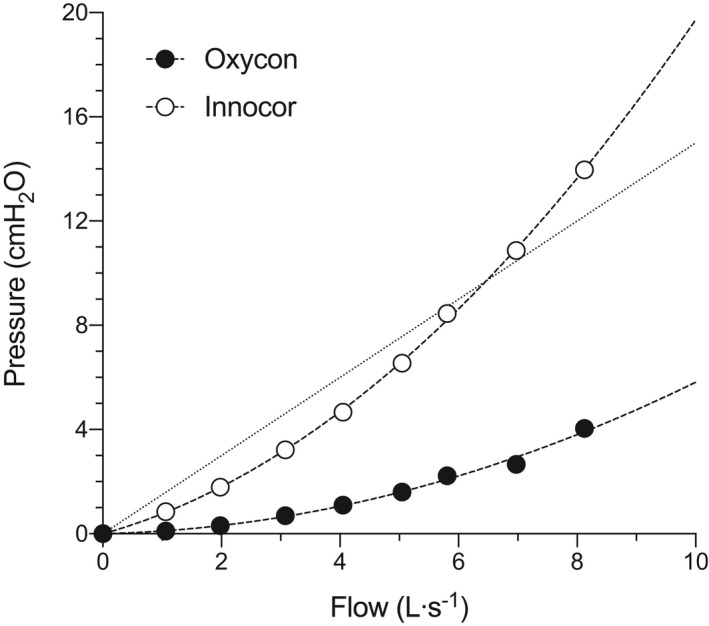
pressure x flow relationship of the Oxycon (closed circles) and the Innocor (open circles) obtained from bench tests using constant airflows of different velocities and pressure transducers. The dotted line represents airflow resistance of 1.5 cmH_2_O∙L^−1^∙s^−1^

The Innocor system (Innovision, Odense, Denmark) is a metabolic cart that uses a pneumotachometer with a low‐resistance screen to measure flow (0.3 cmH_2_O∙L^−1^∙s^−1^ at 5 L.s^−1^, without a bacterial filter) during spirometry. The measurement of gas exchange during ergospirometry requires an additional valve, which further increases resistance, thus placing the device at the upper range of what is considered acceptable by current guidelines. Moreover, the Oxycon (Oxycon Pro, Jaeger, Höchberg, Germany) uses a low‐resistance flat fan (called a triple‐V sensor) to measure airflow, with resistance in the range of ~1 cmH_2_O∙L^−1^∙s^−1^ at an airflow of 14 L∙s^−1^. A detailed view of both set‐ups is shown in Figure 1. Internal bench testing in our laboratory (Figure 2) showed that under constant flow the level of resistance provided by both equipment when assembled as instructed by the manufacturers’ for gas exchange measurements is substantially different, but nonetheless, the Innocor still complies with the stricter guidelines up to a flow of ~7L∙s^−1^, typically seen when trained men are requested to exercise maximally (Mcclaran et al., 1999).

The aim of the present investigation was, therefore, to investigate whether incremental exercise tests performed with the two different metabolic carts of varying resistance to airflow would result in similar values for breathing pattern, gas exchange, and exercise performance in young, healthy male individuals. We hypothesized that tests using the Innocor system would result in higher values of mouth pressure at high exercise intensities, significantly impairing maximal exercise performance.

## 2 METHODS

### 2.1 Ethical approval

Prior to any experiments, participants were thoroughly informed about the methods used in the study and gave their written informed consent. This study was approved by the local ethics committee (2016‐N‐65), and was conducted in accordance with the latest declaration of Helsinki, except for registration in a database.

### 2.2 Study design and overview

Sixteen healthy men (Table 1) The participants reported to the laboratory on two different occasions, separated by at least 72 h, and on each visit two maximal incremental tests (one with the Innocor and one with the Oxycon Pro in randomized, counter‐balanced order) were performed on a cycle ergometer Ergoline 900, Ergoline, Blitz, Germany, with 90 min of rest between tests. At the beginning of the first visit, lung function and respiratory muscle strength were assessed.

**TABLE 1:** Participants’ characteristics

	Participants (n = 16)
Age (years)	25 ± 1
Height (cm)	180 ± 6
Weight (kg)	73.5 ± 5.8
FVC (L)	6.0 ± 0.7
FVC (% pred)	106.3 ± 10.8
FEV_1_ (L)	4.9 ± 0.5
FEV_1_ (% pred)	103.5 ± 9.7
PEF (L∙s^−1^)	10.5 ± 0.8
PEF (% pred)	103.9 ± 7.7
MVV_12_ (L∙min^−1^)	179.5 ± 21.9
MVV_12_ (% pred)	115.4 ± 15
MIP (cmH_2_O)	130 ± 25
MEP (cmH_2_O)	173 ± 35

Values are means ± SD.

Abbreviations: % pred, percent of the predicted value; FEV_1_, forced expired volume in 1 s; FVC, forced vital capacity; MEP, maximal expiratory pressure; MIP, maximal inspiratory pressure; MVV_12_, maximal voluntary ventilation averaged from a 12 s maneuver; PEF, peak expiratory flow.

### 2.3 Procedure and experimental set‐up

#### 2.3.1 Lung function and respiratory muscle strength

Lung function was assessed according to standard procedures (Miller, 2005) using a calibrated flow meter (Oxycon Pro), and using available reference values (Brändli et al., 1996; Quanjer et al., 1993, 2013). Maximal inspiratory pressure (MIP, at residual volume) and maximal expiratory pressure (MEP, at total lung capacity) were measured using a handheld device (MicroRPM, MicroMedical, Kent, UK) according to current guidelines and reference values (Gibson et al., 2002; Wilson et al., 1984).

#### 2.3.2 Incremental exercise test

Prior to each test, the respective metabolic carts were calibrated according to the manufacturers’ instructions. Participants used the same mouthpiece and nose‐clip with both metabolic carts. The Innocor set‐up (standard, as recommended by the manufacturer) included a bacterial filter (PALL Pro‐Tec PF30‐S, PALL Corporation, NY 28 mm internal diameter, 40 ml dead‐space) connected to the mouthpiece. The filter was connected to the flowmeter (differential pressure method, using a mesh screen), which in turn was connected to the L‐shaped respiratory valve unit of the Innocor, the latter containing a disposable silicon insert (103 ml dead‐space). The Oxycon Pro set‐up consists of connecting the face‐mask to the Triple‐V sensor, a short tube with 2.7 cm of internal diameter with an in‐series flat fan (35 ml dead‐space). The tube contains a metallic sputum catcher which guards the spinning part and is estimated to cover about 25% of the available area (Schoffelen et al., 2019). Both metabolic carts use a sample line behind the volume sensors to drive some of the inspired/expired air into the analyzers.

The incremental test started at 100 W for 2 min and the load was increased subsequently by 30 W every 2 min. Participants were requested to choose a comfortable cadence in the range of 60–80 rpm and keep it constant throughout this and the following tests. The test continued until subjective exhaustion or until the cadence could not be maintained with ±3 rpm despite strong verbal encouragement.

During the tests, gas exchange and heart rate were continuously monitored. At the end of each 2‐min stage and at exhaustion, participants were asked to rate their perception of breathlessness, respiratory, and leg exertion using a 20‐cm visual analog scale (VAS) without verbal clues, with values subsequently transformed to a range between 0 and 10 points (Renggli et al., 2008). Twenty microliters of arterialized capillary blood were drawn from an earlobe every 2 min to assess blood lactate concentration ([La^−^]) (BIOSEN C_line Sport^®^, EKF‐diagnostic, Industrie‐Elektronik, Barleben, Germany).

Mouth pressure was continuously monitored using pressure transducers (DP45‐34, Validyne, Northridge, CA, USA). The raw data were collected (5000 Hz, PowerLab; ADInstruments, Sydney, Australia) and stored for subsequent analysis (LabChart v8.0, ADInstruments).

### 2.4 Data analysis and statistics

Peak power output (PPO) was determined as the power at the last completed stage plus the fraction of the final stage multiplied by 30. Peak oxygen consumption (V̇O_2peak_) was determined as the highest value during a test using a 30‐s moving average (10‐s bins), and end‐exercise values for physiological parameters were determined as the average of the final 30 s of each test. The ventilatory threshold was determined according to the V‐slope method (Beaver et al., 1986), and the lactate threshold was determined using the D‐max method (Morton et al., 2012).

Mouth pressure signals were lowpass filtered (50 Hz) to reduce noise, and exported in 0.1‐s bins to Microsoft Excel files. Airflow resistance was then estimated using the relationship between pressure and flow obtained from the bench tests. Values were then averaged in a similar fashion to the gas exchange parameters for analysis.

For each participant, the duration of the shorter of the four tests was deemed isotime and tests were thereafter divided into 10 intervals of equal duration (i.e. 10% of isotime), and the final minute of each interval was used for subsequent analysis. End‐exercise values were considered as the last 30 s of each test. Data for [La^−^], breathlessness, respiratory and leg exertion were compared for each test stage until 220 W (the final common intensity for all participants) and at end‐exercise.

All data are presented as means ± SD and 95% confidence intervals of differences (95% CI) where applicable. After an initial two‐way analysis of variance (ANOVA) had shown no main effects of test day (first vs. second, *F*_(1,9)_ = 0.004, *p* = 0.946) and test order (first vs. second test of a given day, *F*_(1,9)_ = 0.806, *p* = 0.397) for time to exhaustion and V̇O_2peak_ (data not shown), the data from each pair of tests for a given metabolic cart were averaged for further comparisons. Subsequent analyses were performed using 2‐way ANOVAs (equipment and time), with Sidak's *post hoc* test. Comparisons of time to exhaustion and end‐exercise flow resistance were performed using Student's t tests for dependent samples (2‐tailed). Statistical significance was set at α < 0.05, and all tests were performed using Prism 8.0 (GraphPad, La Jolla, CA).

## 3 RESULTS

### 3.1 Respiratory resistance and maximal exercise capacity

**FIGURE 3:**
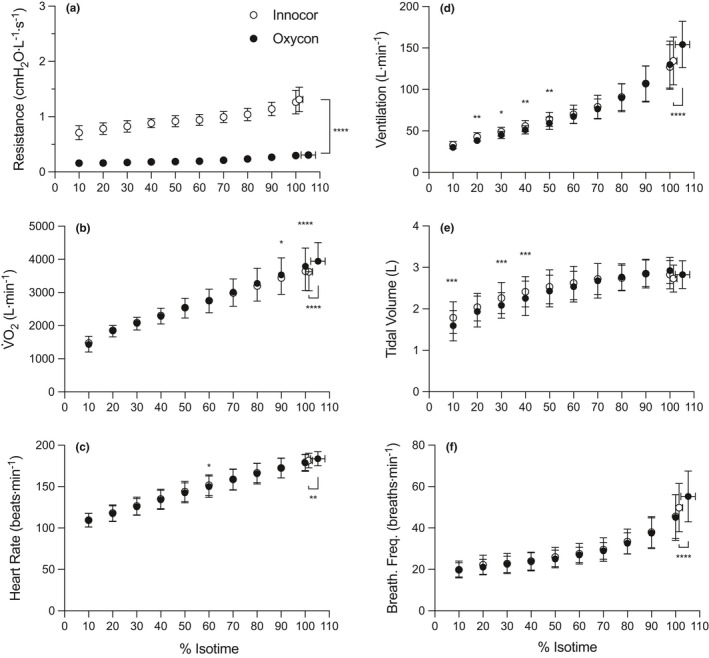
Resistance to expired airflow (a), oxygen uptake (b), heart rate (c), pulmonary ventilation (d), tidal volume (e) and breathing frequency (f) for tests performed with the Innocor (open circles) and Oxycon (closed circles). Data are expressed as % isotime. *p < 0.05, **p < 0.01, ***p < 0.001, ****p < 0.0001 between equipment for a given time‐point

Resistance to airflow was significantly higher with the Innocor throughout the exercise tests (main effect of equipment, *F*_(1, 15)_ = 922.7, *p* < 0.0001, Figure 3a). End‐exercise values for expiratory airflow resistance were higher for the Innocor (1.3 ± 0.2 vs. 0.3 ± 0.0 cmH_2_O∙L^−1^∙s^−1^, 95% CI 0.9 – 1.1, *p* < 0.0001), even though flow was not different (4.65 ± 1.17 vs. 4.63 ± 0.96 L.s^−1^, 95% CI −0.53 – 0.56, *p* = 0.98). In addition, time to exhaustion was shorter (15.3 ± 3.2 vs. 15.8 ± 3.3 min, 95% CI −0.35 – −0.78, *p* < 0.0001) and V̇O_2peak_ was lower (3764 ± 528 vs. 3964 ± 526 ml∙min^−1^, 95% CI −234 – −401, *p* < 0.0001) in the tests performed with the Innocor compared with the Oxycon.

To ensure the validity of our results with regards to the stricter guidelines, airflow resistance, time to exhaustion and V̇O_2peak_ data were analyzed excluding one participant who showed an airflow resistance >1.5 cmH_2_O∙L^−1^∙s^−1^ at end‐exercise (which occurred in only one of the Innocor tests, but the average remained at 2.0 cmH_2_O∙L^−1^∙s^−1^ for a flow of 6.9 L⋅s^−1^). For the remaining participants, tests with Innocor showed shorter test duration (−0.6 ± 0.4 min, *p* < 0.0001), lower peak work rate (−9 ± 6 W, *p* < 0.001), lower V̇O_2peak_ (−200 ± 228 ml O_2_.min^−1^, *p* = 0.0043) and higher airflow resistance (+1.0 ± 0.1 *p* < 0.0001).

### 3.2 Physiological responses

V̇O_2_ was similar between equipment until 80% of isotime, and thereafter became significantly lower with the Innocor (equipment‐time interaction, *F*_(10, 130)_ = 13.49, *p* < 0.0001, Figure 3b). Heart rate was similar between equipment for the majority of the isotime period, except at 60% of isotime (equipment‐time interaction, *F*_(10, 150)_ = 3.373, *p* = 0.0005) when it was higher with the Innocor (1.6 ± 0.6 beats∙min^−1^, *p* = 0.04). Heart rate at end‐exercise was lower with the Innocor (181.5 ± 8.8 vs. 183.6 ± 8.5 beats∙min^−1^, 95% CI −0.5 – −3.7, *p* = 0.002, Figure 3c).

V̇_E_ was higher with the Innocor between 20 and 50% of isotime (equipment‐time interaction, *F*_(10, 150)_ = 30.96, *p* < 0.0001), but became lower than the Oxycon at end‐exercise (134.3 ± 28.8 vs. 154.2 ± 28.0 L∙s^−1^, 95% CI −16.2 – −23.5, *p* < 0.0001, Figure 3d). Tidal volume (V_T_) was higher with the Innocor at 10, 30 and 40% of isotime (equipment‐time interaction, *F*_(10, 150)_ = 6.832, *p* < 0.0001, Figure 3e), while breathing frequency (f_B_) was only different between equipment at end‐exercise (equipment‐time interaction, *F*_(10, 150)_ = 6.369, *p* < 0.0001, Figure 3f), with lower values achieved with the Innocor (49.8 ± 11.7 vs. 55.2 ± 12.3 breaths∙min^−1^, 95% CI −3.3 – −7.5, *p* < 0.0001, Figure 3f).

**FIGURE 4:**
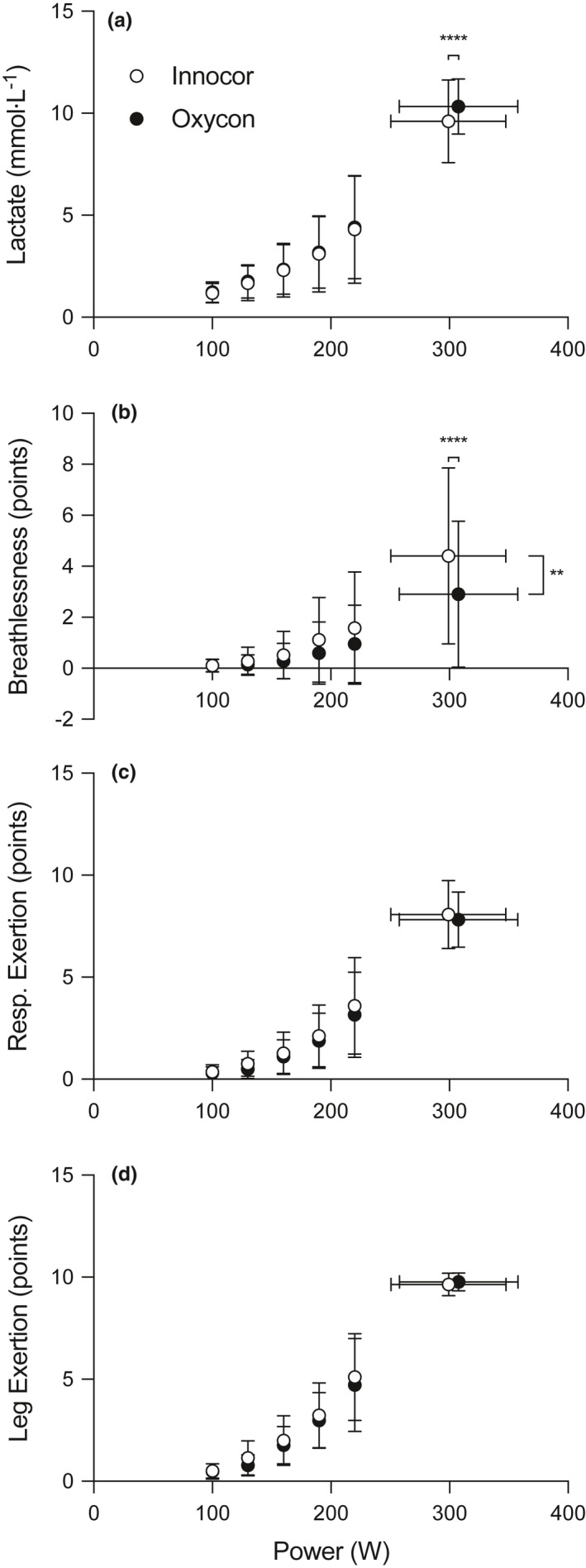
Blood lactate concentration (a), breathlessness (b), respiratory (c) and leg exertion (d) for tests performed with the Innocor (open circles) and Oxycon (closed circles). Data are expressed as % isotime. *p < 0.05, **p < 0.01, ***p < 0.001, ****p < 0.0001 between equipment for a given time‐point

Lactate and ventilatory thresholds did not differ between the two devices (Table 2), regardless of whether the threshold was expressed in absolute power or as a fraction of PPO. [La^−^] at end‐exercise were different between equipment (equipment‐time interaction, F_(5, 70)_ = 4.058, *p* = 0.0027) with lower values with the Innocor (9.6 ± 2.0 vs. 10.3 ± 1.3 mmol∙L^−1^, 95% CI −0.4 – −1.1, *p* < 0.0001, Figure 4a).

**TABLE 2:** Power output at the lactate and ventilatory thresholds

	Innocor	Oxycon	*p*
Lactate Threshold			
Absolute (W)	220 ± 28	226 ± 28	0.07
Relative (%PPO)	75.1 ± 3.5	75.2 ± 4.2	0.91
Ventilatory Threshold			
Absolute (W)	190 ± 39	194 ± 43	0.17
Relative (%PPO)	63.4 ± 7.9	62.7 ± 6.6	0.53

Values are means ± SD. % PPO, percent of peak power output determined during the incremental test.

### 3.3 Perceptual responses

The sensation of breathlessness was higher with the Innocor compared with the Oxycon (main‐effect of equipment *F*_(1, 15)_ = 9.088, *p* = 0.0087), while pair‐wise comparisons only showed a difference at end‐exercise (equipment‐time interaction, *F*_(5, 75)_ = 3.013, *p* = 0.0156), with higher values for the Innocor (4.4 ± 3.5 vs. 2.9 ± 2.9 points, 95% CI 0.6 – 2.3, *p* < 0.0001, Figure 4b). Moreover, there were no differences between equipment at any time‐point for the sensations of respiratory exertion (equipment‐time interaction, *p* = 0.71, Figure 4c) or leg exertion (equipment‐time interaction, *p* = 0.09, Figure 4d).

## 4 DISCUSSION

The primary aim of this study was to test whether using a metabolic cart with higher but acceptable levels of flow resistance would adversely impact performance, physiological and perceptual responses in an incremental exercise to exhaustion in healthy individuals. The most relevant findings are that flow resistance in the upper range of accepted limits i) negatively impacted exercise tolerance and ii) significantly affected several gas exchange parameters during sub‐maximal and maximal exercise.

The level of respiratory resistance created by the Innocor is substantially lower than that used in studies specifically intending to create respiratory resistance (for example 3–5 cmH_2_O∙L^−1^∙s^−1^ in Harms et al. (2000)), but nonetheless, differences in the sensation of breathlessness and V̇_E_ were apparent at end‐exercise, suggesting that resistances around 1.0–1.2 cmH_2_O∙L^−1^∙s^−1^ are sufficient to evoke different metabolic and perceptual responses during near‐maximal exercise and therefore impair exercise capacity. Loading the inspiratory or expiratory muscles decrease voluntary force production during simultaneous contraction of the quadriceps (Turner & Jackson, 2002) and increases the perception of breathlessness and leg fatigue during cycling (Harms et al., 2000), potentially explaining the similar leg exertion at end‐exercise between conditions despite the lower power output with the Innocor. Additionally, maximal perception of breathlessness, as seen in two of our participants with the Innocor and none with the Oxycon, has been shown to create sufficient discomfort to be listed as the reason for exercise termination (Kayser et al., 1997). This would be in line with the lower V̇O_2peak_ reached with the Innocor, possibly indicating that participants chose to terminate the test due to respiratory discomfort or other unpleasant sensations rather than reaching true maximal cardiorespiratory capacity.

Despite the higher level of breathlessness and airflow resistance, participants reported similar respiratory exertion between the Innocor and the Oxycon throughout the exercise tests. The sensation of respiratory muscle exertion has been shown to be a function of the peak pressure produced, akin to the sensation of lifting weights (Killian et al., 1982), and is further modulated by the actual ventilatory drive produced by a given condition (Burdon et al., 1982). A higher sense of respiratory muscle exertion, therefore, would be expected on a condition in which mouth pressure and breathlessness were higher. However, the decreased f_B_ at end‐exercise with the Innocor might have contributed to lower the sensation of exertion, as previously shown (el‐Manshawi et al., 1986; Killian et al., 1982). Another possibility is that participants might have been unable to fully separate breathlessness from respiratory exertion despite the training given, as also noted by Lansing et al., (2000). Recently it has also been shown that when dyspnea is described as “the sensation of labored or difficult breathing” it can be dissociated from the actual work of breathing (Molgat‐Seon et al., 2018), similar to our breathing exertion results.

Differences in breathing pattern between the two tests, however, were seen already during the first 50% of isotime, when airflow resistance too was different between devices. Since participants wore the same mouthpiece and nose clips on both devices, any differences between equipment were necessarily caused by the extra resistive load to airflow and not by breathing through different systems (Perez & Tobin, 1985). The higher V̇_E_ and V_T_ with the Innocor might simply reflect greater awareness of breathing, which was later overridden by physiological mechanisms. At end‐exercise, moreover, V̇_E_ and f_B_ were lower with the Innocor compared to the Oxycon. Although respiratory muscle fatigue could be hypothesized as a potential cause for this observation, the occurrence of respiratory muscle fatigue disputed during incremental tests (Ozkaplan et al., 2005; Romer et al., 2007), at least without using additional respiratory loads. Apart from the possibilities that participants were unable to reach their desired V̇_E_ due to excessive resistance or the occurrence of respiratory muscle fatigue, the lower V̇_E_ could have resulted from the lower time to exhaustion, lower [La^−^] and likely higher blood pH, as V̇_E_ was similar between equipment at 100% of isotime. Furthermore, the ventilatory and lactate threshold happened at similar time points between conditions, suggesting a similar acid‐base state.

Even though respiratory muscle fatigue itself is an unlikely explanation for the decreased end‐exercise V̇_E_ and lower performance with the Innocor, the increased resistance might still have affected performance. Muscle sympathetic nerve activity (MSNA) increases progressively during cycling with expiratory loading, with differences noted already after 1 min of exercise, when fatigue is unlikely to have occurred (Katayama et al., 2015). Likewise, reductions in limb blood flow and an increase in limb vascular resistance were shown within the 1^st^ min of inspiratory muscle loading (Sheel et al., 2001). Assuming the increase in work of breathing caused by the flowmeter of the Innocor was of sufficient magnitude to increase leg MSNA and deviate blood flow away from the exercising limbs via the respiratory muscle metaboreflex (Dempsey et al., 2008), this would clearly have negative consequences for performance, as at the end of an incremental test blood flow to the limbs is maximal (Wagner, 2011).

The interplay between increased respiratory resistance, peak workload, and gas exchange during the incremental exercise tests with and without added respiratory resistance is not straightforward. As V̇O_2max_ is a finite resource, it is clear that higher values should not be expected with added respiratory resistance during the tests; rather, a portion of the O_2_ that would normally be taken by the legs is likely used up by the respiratory muscles, resulting in no net difference for pulmonary V̇O_2_. At the same time, the work of breathing itself might not have been higher with the added resistance, as V̇_E_ was lower at peak exercise with the Innocor and the relationship between V̇_E_ and work of breathing becomes steeper at very high VE. Evidently, for any given V̇_E_ the work of breathing (and therefore O_2_ consumption by the respiratory muscles) would be increased with extra respiratory resistance, but the influence of such increase on pulmonary V̇O_2_ depends on participants being able to achieve similar workloads and work of breathing. In our study, the additional resistance prevented participants from reaching a similar W_max_, which together with a lower V̇_E_ seems to have affected the scale toward a lower pulmonary V̇O_2_ with added respiratory resistance, therefore, underestimating the aerobic fitness of participants.

### 4.1 Limitations

It would have been interesting to compare the O_2_ cost of breathing between the devices. In the present study, only whole body V̇O_2_ was measured and any increases in respiratory muscle V̇O_2_ during submaximal exercise would need to surpass the variability created by comparing data from different tests and using different equipment.

We studied young, healthy male participants only. While the differences in equipment would persist regardless of participants, any other group producing smaller flows during intense exercise would likely be less affected by the higher resistance. Nonetheless, the O_2_ cost of ventilation accounts for up to 13%–15% of V̇O_2peak_, (Aaron et al., 1992), and is higher in women than in men (Dominelli et al., 2015), which could potentially exacerbate the effects of higher resistance to airflow. Moreover, women seem to have higher diaphragmatic fatigue resistance (Welch et al., 2018a), but once fatigue is present the effects on subsequent exercise are similar to that observed in men (Welch et al., 2018b).

A key aspect of this study was to compare the effects of different equipment on exercise performance. This, moreover, means that different equipment were used to assess gas exchange data in the different tests. Although we performed internal cross‐validation of the devices (data not shown) the possibility that some differences are due to technological error rather than physiological in nature cannot be completely excluded. For instance, the higher dead space on the Innocor might have led to increased V_T_ and V̇_E_, although differences in dead space were small.

## 5 CONCLUSION

The present work shows how a small increase in respiratory resistance, still within recommended guidelines, may affect breathing mechanics and exert a negative effect on exercise capacity, especially in healthy young male individuals who can produce large V̇_E_ and flow values during intense exercise. Therefore, the present results indicate the need to revisit guidelines for devices used in ergospirometry.

## CONFLICT OF INTERESTS

The authors have no interests to disclose.

## AUTHOR CONTRIBUTIONS

FGB and CMS designed the study. FGB, JC, ER, and CMS approved the study design. FGB, ER, and JK collected and analyzed the study data. All authors contributed to the interpretation of the data. FGB drafted the initial manuscript. All authors revised and approved the final manuscript.

## ETHICAL STATEMENT

This study was approved by the local ethics committee (2016‐N‐65) and was conducted in accordance with the latest declaration of Helsinki, except for registration in a database.

## Data Availability

The data sets generated during and analyzed during the current study are available from the corresponding author on reasonable request.
